# Severely malnourished children with a low weight-for-height have a higher mortality than those with a low mid-upper-arm-circumference: I. Empirical data demonstrates Simpson’s paradox

**DOI:** 10.1186/s12937-018-0384-4

**Published:** 2018-09-15

**Authors:** Emmanuel Grellety, Michael H. Golden

**Affiliations:** 1 Research Center Health Policy and Systems - International Health, School of Public Health, Université Libre de Bruxelles, Bruxelles, Belgium; 2 Department of Medicine and Therapeutics, University of Aberdeen, Aberdeen, Scotland

**Keywords:** Nutrition, Acute malnutrition, Severe acute malnutrition, SAM, Mid-upper-arm circumference, MUAC, Weight-for-height, WHZ, Mortality, Case fatality rate, Wasting, Oedema, Kwashiorkor, Diagnosis, Simpson’s paradox, Mathematical coupling, Child, Human, Meta-analysis

## Abstract

**Background:**

According to WHO childhood severe acute malnutrition (SAM) is diagnosed when the weight-for-height Z-score (WHZ) is <−3Z of the WHO_2006_ standards, the mid-upper-arm circumference (MUAC) is < 115 mm, there is nutritional oedema or any combination of these parameters. Recently there has been a move to eliminate WHZ as a diagnostic criterion on the assertion that children meeting the WHZ criterion are healthy, that MUAC is universally a superior prognostic indicator of mortality and that adding WHZ to the assessment does not improve the prediction; these assertions have lead to a controversy concerning the role of WHZ in the diagnosis of SAM.

**Methods:**

We examined the mortality experience of 76,887 6–60 month old severely malnourished children admitted for treatment to in-patient, out-patient or supplementary feeding facilities in 18 African countries, of whom 3588 died. They were divided into 7 different diagnostic categories for analysis of mortality rates by comparison of case fatality rates, relative risk of death and meta-analysis of the difference between children admitted using MUAC and WHZ criteria.

**Results:**

The mortality rate was higher in those children fulfilling the WHO_2006_ WHZ criterion than the MUAC criterion. This was the case for younger as well as older children and in all regions except for marasmic children in East Africa. Those fulfilling both criteria had a higher mortality. Nutritional oedema increased the risk of death. Having oedema and a low WHZ dramatically increased the mortality rate whereas addition of the MUAC criterion to either oedema-alone or oedema plus a low WHZ did not further increase the mortality rate. The data were subject to extreme confounding giving Simpson’s paradox, which reversed the apparent mortality rates when children fulfilling both WHZ and MUAC criteria were included in the estimation of the risk of death of those fulfilling either the WHZ or MUAC criteria alone.

**Conclusions:**

Children with a low WHZ, but a MUAC above the SAM cut-off point are at high risk of death. Simpson’s paradox due to confounding from oedema and mathematical coupling may make previous statistical analyses which failed to distinguish the diagnostic groups an unreliable guide to policy. WHZ needs to be retained as an independent criterion for diagnosis of SAM and methods found to identify those children with a low WHZ, but not a low MUAC, in the community.

**Electronic supplementary material:**

The online version of this article (10.1186/s12937-018-0384-4) contains supplementary material, which is available to authorized users.

## Background

About 19 million children are estimated to have severe wasting, of whom about half to one million die each year [1]. These estimates were made from prevalence data using weight-for-height (WHZ) as the single criterion. As the deaths related to a low mid-upper-arm-circumference (MUAC) or nutritional oedema (kwashiorkor) were not included in these estimates the actual prevalence is much higher than this estimated burden. Furthermore, although the prevalence may have been overestimated with respect to WHZ [2], the incidence was not taken into account; this would increase the annual burden much more substantially [3]. Whatever the actual magnitude it is clear that severe acute malnutrition (SAM), with other nutritional insults, are major neglected conditions leading to death and poor development of children globally and as such constitute a critical public health priority. The criteria used to define SAM have a crucial effect upon all aspects of the condition.

Not only assessments of the numbers of children affected but also their individual eligibility for treatment is affected by the criteria used to define SAM. These criteria have changed repeatedly over the years so that different numbers and degrees of severity have characterised those designated as having SAM. These schemes initially included those based upon weight-for-age, introduced by Gomez [4] and adopted by The Wellcome Trust [5], as the basic parameter [6]. Later weight-for-height was suggested [7] and forms a classification by Waterlow [8] to differentiate underweight children (weight-for-age) into those that are light because they are wasted (weight-for-height, WHZ) from those that are small because they are stunted (height-for-age). Wasting and stunting are thought to represent acute and chronic malnutrition and to be appropriately treated, respectively, with an acute intervention to reverse the wasting and prevent death or long term support to the child and family to permit sustained improvement of growth and development. The normal references to which the malnourished children are compared have also been refined successively from the Baldwin-Wood [9], Harvard [10], NCHS [11], CDC_2000_ [12] and more recently to the WHO_2006_ references [13]. The WHO_2006_ references are now promulgated as being standards, rather than references, to which all children should aspire for optimal health [14]. They have rendered all other references obsolete.

Since Waterlow’s classification [8], SAM has been defined as children having a low WHZ and/or nutritional oedema. More recently WHO has endorsed the additional criterion of a low absolute MUAC as an independent criterion to classify children with SAM [15]. Therefore the universal definitions of childhood SAM now mandated by WHO are a WHZ of <−3Z of the WHO_2006_ standards or an absolute MUAC of < 115 mm or nutritional oedema, or any combination of these three criteria.

Because of its simplicity, ease of use and relative cheapness as a diagnostic tool MUAC has been readily taken up to screen children for SAM in the community and elsewhere [16]. It can even be used by mothers themselves [17]. The development of a therapeutic food suitable and safe to give at home [18] has led to a revolution in the care of SAM children [19, 20], to scaling up of treatment programs (SUN movement) and “coverage” assessed by the proportion of SAM children diagnosed by MUAC in the community that are receiving treatment [21]. Thus, MUAC has been widely adopted by many agencies and some governments as the preferred criterion for diagnosis of SAM and is used to select children for treatment from the community and health facilities in accordance with WHO recommendations [22]; these agencies no longer assess WHZ and now run “MUAC only” programs. Children admitted by MUAC show as good a response to treatment in the community as those admitted by the WHZ criterion [23] particularly if they have a good appetite, are uncomplicated and are relatively close to the 115 mm threshold. Community programs prevent the milder forms of SAM from deteriorating further and developing complications and have enabled many children to access treatment at home who would otherwise have remained untreated.

Although the prevalence of SAM (and moderate acute malnutrition - MAM), is about the same in nutritional surveys when diagnosed by MUAC and WHZ, different children are identified by the two criteria with a considerable discordance in individual countries [24–41]. We previously collected data from representative community surveys of children from 47 countries to assess the degree of overlap for SAM and MAM by the two anthropometric criteria, to examine the external validity, the scale and direction of discordance and how it varied by country [42]. We found that the two criteria performed quite differently in the various countries and regions, with some diagnosing most SAM children with MUAC and others nearly all SAM children with WHZ. There was no satisfactory explanation for this phenomenon (see [42] for discussion). The mean overlap for SAM (children fulfilling both the WHZ and MUAC criteria) was 16.5%, so that more than 80% of children in the community had SAM by one or the other but not by both criteria. About 45% of the children fulfilled the WHZ definition for SAM but not the MUAC criterion; i.e. they were identified by WHZ alone because they had an absolute MUAC of over 115 mm. As the two diagnostic parameters select different children we proposed that both MUAC and WHZ should continue to be used routinely to identify those children who should receive treatment.

This suggestion led to a direct criticism from Briend et al. [43] who maintain that only MUAC should be used to identify severely malnourished children, that this is a public health priority and nothing should divert resources from universal use of MUAC as the only criterion for diagnosing and selecting children. The position taken by Briend et al. appears to have widespread approval shown by his numerous co-authors and support from humanitarian agencies and donors. However, Briend et al’s proposal has led to a controversy among the humanitarian and nutritional community concerning whether WHZ should or should not be abandoned as a criterion for the diagnosis of SAM. A major assertion justifying their point of view is that children with a low WHZ are relatively healthy [44–50] and therefore are not in need of treatment. Briend et al. [43, 46] also contend 1) that WHZ can be abandoned on the grounds that MUAC has repeatedly been shown to be a better indicator of mortality than WHZ, based solely on comparison of receiver operating characteristic curves (ROC) to predict long-term, all-cause mortality risk, 2) that they only have a low WHZ because their legs are relatively long, 3) that the two criteria are proxies for each other and 4) that when children satisfy both criteria their mortality rate is not additive, but that MUAC mortality is always higher than that of WHZ [43, 46, 49–51], and 5) that the addition of WHZ to MUAC does not increase the prognostic sensitivity or specificity of death prediction [51]. These contentions have each been rebutted [52] (rebuttal follows after [43]). At stake is the fate of the 45% of children with SAM by WHZ but not by the MUAC; if they are indeed relatively healthy at a low risk of death then dropping the use of WHZ may have merit, however, if they are at a high risk of death such a policy would lead to a large proportion of SAM children being denied treatment.

The purpose of this study is to address the controversy by examining the relative mortality rates of children who have SAM by the three different WHO recommended criteria; a WHZ of <−3Z using the WHO_2006_ standards, a MUAC of < 115 mm, and nutritional oedema (kwashiorkor and marasmic-kwashiorkor), each separately as well as the various combinations of the three criteria.

Our a priori hypotheses were 1) that children with SAM by MUAC-only and WHZ-only both have a substantial mortality risk, 2) that the two conditions are additive so that children satisfying both criteria have an augmented mortality risk and 3) that nutritional oedema further augments the risk of death. We did not hypothesise that SAM by MUAC-only or WHZ-only would have a higher mortality rate in older or younger children. On one hand younger children are more likely to have a MUAC < 115 mm but also have an inherently higher mortality rate, on the other hand an older child who fulfils the MUAC criterion will be more severely malnourished. Thus, we a priori determined to examine relative mortality by age group.

## Methods

We re-analysed data from in-patient treatment facilities (IPFs), out-patient treatment programs (OTPs) and supplementary feeding centres (SFCs) to determine the mortality rates associated with combinations of the different diagnostic criteria: MUAC, WHZ and oedema using the WHO_2006_ recommended criteria that now define marasmus, kwashiorkor and marasmic-kwashiorkor.

In order to have a sufficient number of deaths, admission weight, height, MUAC, oedema, age, sex and outcome data were collected from patients that had been treated for SAM from three sources: 1) Therapeutic feeding centres and hospitals in African countries; these children, with complicated SAM were all under intensive daily care and are collectively referred to as being treated in in-patient facilities (IPFs); 2) Children with uncomplicated SAM with a reasonable appetite were treated in out-patient therapeutic programs (OTPs), and followed weekly; and, 3) Children initially classified as having MAM who were given take-home supplementary food and followed either every 2 weeks or monthly at supplementary feeding centres (SFCs).

All the data were retrospective and involved only children that were being treated using standard WHO therapeutic protocols [53] for earlier studies and updated versions for later studies [54] and for those treated as outpatients [55]. Although the treatment given in each type of program was different, the treatment given in each mode of treatment was standardised according to WHO and updates, derivative National Guidelines on Integrated Management of Severe Malnutrition and derivate Non-Governmental Organisations’ (NGO) guidelines. There were only minor differences between the documents. Most programs were carried out by International NGOs so that cross facility and country treatment was the same and the supervisory staff had had the same training at head-quarter level. A few were conducted under the auspices of UNICEF and again followed standard treatment guidelines.

Each child’s individual data had been recorded on forms designed specifically for the management of the severely malnourished according to the guidelines (they are not designed for research purposes). During original data collection these were verified by checking with the centres’ admission’s registry.

The data from all the IPFs, OTPs and SFCs were combined to give three separate datasets of individual patients admitted for one of the three modes of treatment. This was because individual facilities did not contain sufficient deaths to allow for meaningful statistical analysis. Some of the IPF data has already been reported [56]; others were obtained personally for the purpose of program evaluation during visits by MHG and Dr. Yvonne Grellety for National Governments, UNICEF and NGOs; most were from ongoing therapeutic programs by various NGOs.

SFCs should not have recruited any SAM children as these are specifically designed for treatment of MAM children only. However, with the introduction of the WHO_2006_ standards some children who had been classified as moderately malnourished using the NCHS reference or a MUAC cut-off of < 110 mm now satisfied the criteria for SAM with the WHO_2006_ standards and the introduction of a higher MUAC cut-off point as admission criteria for SAM. The data for those children, re-classified as SAM, were abstracted from the SFC data and constitutes a separate dataset for the purpose of this study. As the difference between the NCHS and the WHO standards affect mainly children below about 72 cm in height when NCHS <−3Z was used and all children when < 70% of the NCHS median was used as the admission criterion, the reclassification mainly selected younger children. These children would all have milder forms of SAM as their WHZ fell into the “tramlines” between the two references; those with more serious illness would have been treated in the OTP or IPF.

The individual datasets from each centre and program had been recorded by the authors or by the staff of the NGO running the program using either SPSS, Epi-info or Excel (various versions). They were all transferred to ENA-for-SMART software [57] and their WHZ computed using the WHO_2006_ gender specific standards.

**Fig. 1:**
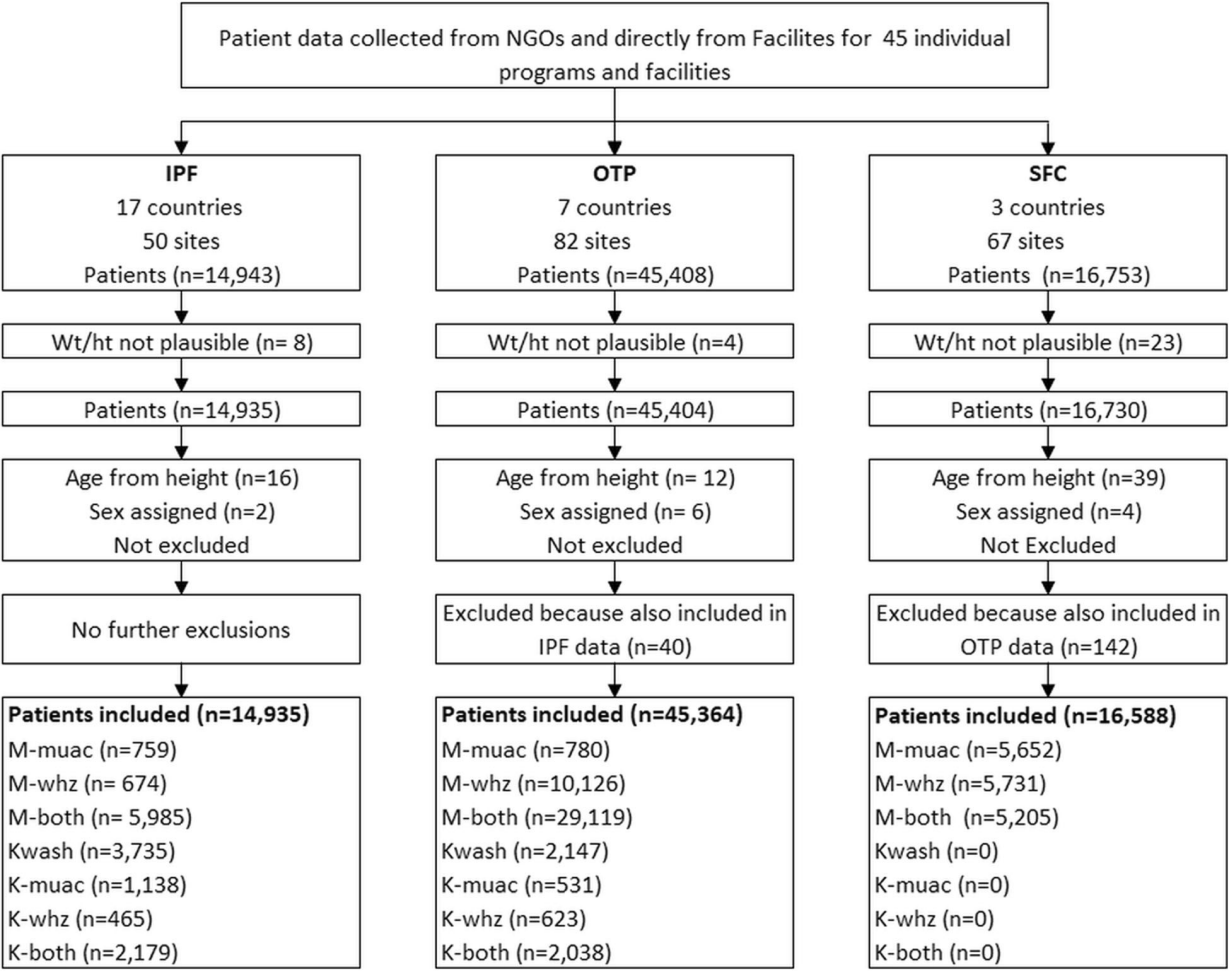
Flow chart of analyses of admissions for treatment of Severe Acute Malnutrition in Africa. *NGOs* Non-Governmental Organizations; *IPF* In-patient Facility (Hospital. Therapeutic Feeding Center); *OTP* Out-patient Treatment Program (Home treatment); *SFC* Supplementary Feeding Center; *Wt/ht* Weight or height; *M-muac* MUAC < 115 mm with WHZ ≥ −3Z and no oedema (marasmus by MUAC only); *M-whz* WHZ < −3Z with MUAC ≥115 mm and no oedema (marasmus by WHZ only); *M-both* MUAC < 115 mm & WHZ < −3Z and no oedema (marasmus by both diagnostic criteria); *Kwash* nutritional oedema/Kwashiorkor without meeting either MUAC or WHZ criteria; *K-muac* oedema & MUAC < 115 mm with WHZ ≥ −3Z (marasmic kwashiorkor by MUAC only); *K-whz* oedema & WHZ < −3Z with MUAC ≥115 mm (marasmic kwashiorkor by WHZ only); *K-both* oedema & MUAC < 115 mm & WHZ < −3Z (marasmic kwashiorkor by both diagnostic criteria)

All children that did not meet at least one of the criteria for SAM, were below 6 or above 60 months or the data for weight, height, MUAC or outcome was absent were excluded. The data were examined for gross errors of recording (such as a child with a height of 10 cm, a weight of 30 kg or a MUAC of 50 mm) which could not occur in children from 6 to 60 months; these records were also excluded. A flow-chat of the data handling and cleaning is given in Fig. 1. No records were excluded on the basis of either WHO or SMART flagging of extreme values; it was assumed that children who were below the cut-off points used during data-cleaning for survey analysis would still fall below the cut-off points for SAM and so were correctly categorised.

As no data were being analysed that depended upon accurate recording of age, where age was not recorded they were retained in the analysis if their heights were between 55 and 115 cm and assigned an age according to their height so that breakdown of the children’s outcome into broad age groups would not be biased (71 children: less than 0.01% of all patients). Where oedema was not recorded they were assumed to be oedema free. Oedema status was not recorded for any of the children in SFC as the presence of oedema is a criterion for direct admission to SAM treatment programs; all these children were assumed to be oedema free. Where sex was not recorded they were assigned a sex at random (12 children).

In keeping with intention-to-treat practice, for each dataset, children that defaulted were retained in the analysis as they were at risk of death prior to their default and most deaths from malnutrition occur early after admission before most defaults occur and children in extremis are less likely to default (the numbers of defaulting children are given in Table 1. Children recorded as failing to respond to treatment were retained. The outcome of non-responders and defaulters after quitting the service is unknown; there was no recorded follow-up of such children in any of the programs. Some of the children in the OTP were recorded as “other”; these were children who moved out of the catchment area, were transferred to a different OTP or started their treatment in the IPF and continued treatment successfully in the OTP. They were retained in the analysis (they were not included in the IPF data).

Children from the SFC who were recorded as being transferred to an OTP or IPF were excluded if the data from the receiving treatment facility were available, otherwise they were included. Similarly, for the OTP, children that were transferred to the IPF were excluded from the analysis if the corresponding IPF data were available, otherwise they were included (i.e. no child was counted in both programs). Children that were transferred to other facilities from the IPF were included in the analysis and assumed to have survived; they were mostly children sent for surgery, tuberculosis or other specialised treatment after their initial severe malnutrition has been successfully managed.

The absolute numbers of children admitted in one of the following categories was determined for each data set divided by whether they left the program alive or dead. Children having:

1. MUAC < 115 mm as the only criterion for admission (WHZ ≥ −3Z, no oedema) - (M-muac);
2. WHZ < −3Z as the only criterion for admission (MUAC ≥115 mm, no oedema) - (M-whz);
3. MUAC < 115 mm and WHZ < −3Z (no oedema) - (M-both);
4. oedema with MUAC ≥115 mm and WHZ ≥ −3Z - (Kwash);
5. oedema with MUAC < 115 mm and WHZ ≥ −3Z - (K-muac);
6. oedema with MUAC ≥115 mm and WHZ < −3Z - (K-whz);
7. oedema with MUAC < 115 mm and WHZ < −3Z - (K-both).

The abbreviations given in parenthesis are used where M- indicates marasmus, K- indicates kwashiorkor/oedematous malnutrition, “muac” is used when the *only* anthropometric criterion is a MUAC of < 115 mm, “whz” when the *only* anthropometric criterion is a WHZ < −3Z WHO_2006_, and the suffix “both” when the child has both a MUAC < 115 mm and a WHZ < −3Z.

The data for non-oedematous and oedematous children were analysed separately. Those children who were alive at exit from the program compared to those that had died were analysed by 2 × 3 and 2 × 4 Chi-squared analysis respectively. The post-hoc individual comparisons were made using the Marascuilo procedure [58, 59]. The complete data were also analysed using a 2 × 7 chi-squared analysis with post hoc Marascuilo comparisons to confirm the significance of comparisons of interest with increased degrees of freedom (data not presented as they include a number of comparisons that were not considered a priori).

The individual comparisons were then re-tested by grouping all the children with a low MUAC (i.e. M-muac + M-both) to give a count of all the children admitted who had a low MUAC – designated as “ALL-muac”. Similarly all children with a low WHZ (M-whz + M-both) were combined to be analysed as “ALL-whz”. This was repeated with the K-muac, K-whz and K-both groups and also with the grand total of oedematous and non-oedematous children combined. These additional analyses were made because most of the published literature on comparison of MUAC and WHZ criteria for diagnosis of SAM includes children that are oedematous and most do not distinguish those who have a single deficit from those that have both a low MUAC and a low WHZ (studies reviewed in the companion paper – [60]).

Because we anticipated that there would be a difference in the relative mortality in younger and older age groups all the analyses were repeated using children 6 to < 18, 18 to < 36 and 36 to 60 months of age.

To determine whether there were regional differences in case fatality rates (CFRs) of children who were admitted by MUAC-only or WHZ-only we combined the data from countries within each region of Africa (see Additional file 1: Table S1 for combinations of countries) by treatment program for comparison of the respective mortality risks. The data were analysed by binary meta-analysis using MetaXL version 5.3 [61]. The odds ratios comparing the case fatality rates for children admitted with M-muac v M-whz and also K-Muac v K-whz were compared using Peto’s method [62] of weighting the groups. No adjustment for the quality of each set of data was made. We did not have sufficient access to any potential confounding data to adjust for confounding.

### Ethical statement

This is a secondary analysis of existing anonymous data which had been collected and analysed for programmatic purposes: that is, to audit services, compare the performance with the Sphere standards [63], identify were performance needed improvement and assess case-loads for future staff and product requirement planning. As no individual, location or administrative district could be identified formal ethical clearance was not required.

## Results

**Fig. 2:**
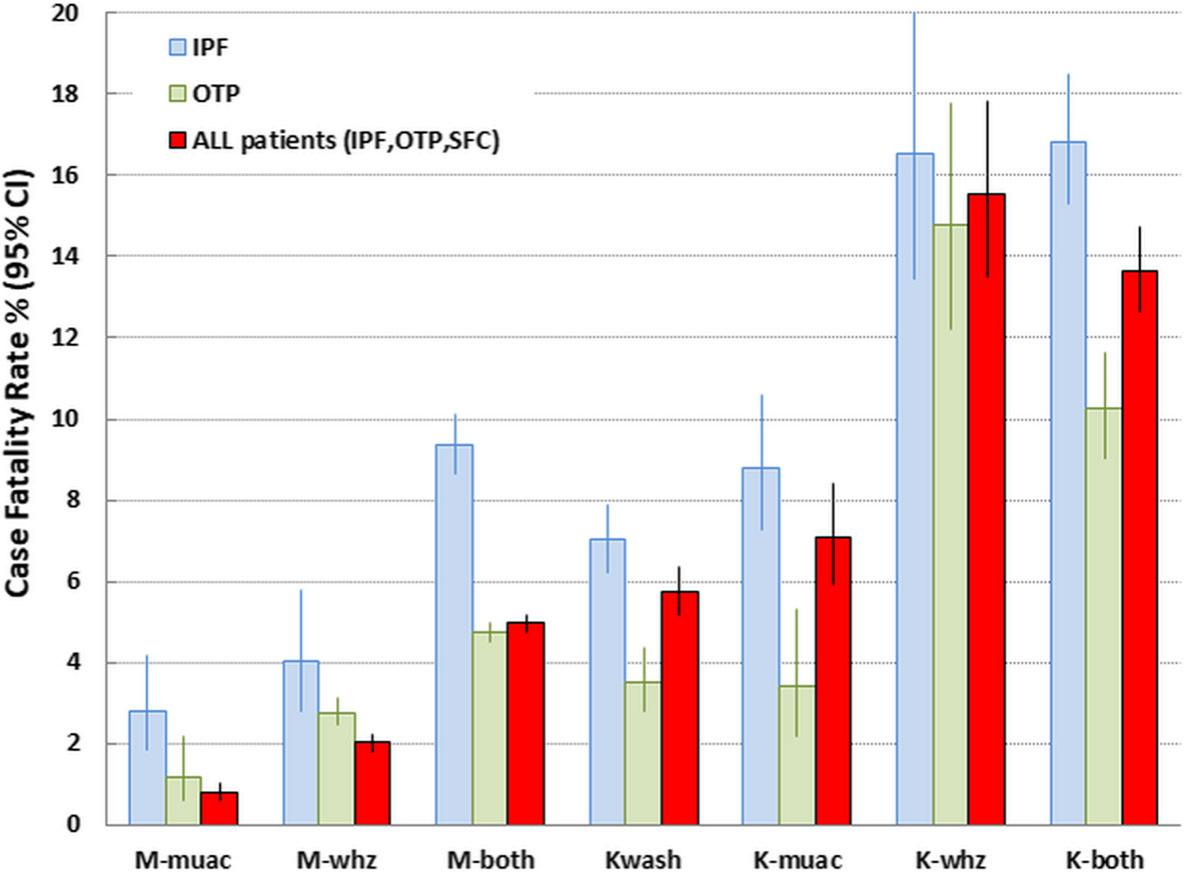
Case-fatality rates of children with SAM aged 6–60 by diagnostic criteria and treatment program. *IPF* In-patient Facility; *OTP* Out-patient Treatment Program; *All patients* refers to the combined totals of children in the IPF, OTP and the SFC (Supplementary Feeding Center); *M-muac* MUAC < 115 mm with WHZ ≥ −3Z and no oedema; *M-whz* WHZ < −3Z with MUAC ≥115 mm and no oedema; *M-both* MUAC < 115 mm & WHZ < −3Z and no oedema; *Kwash* nutritional oedema/Kwashiorkor without meeting either MUAC or WHZ criteria; *K-muac* oedema & MUAC < 115 mm with WHZ ≥ −3Z; *K-whz* oedema & WHZ < −3Z with MUAC ≥115 mm; *K-both* oedema & MUAC < 115 mm & WHZ < −3Z; *CFR* Case fatality rate

**Fig. 3:**
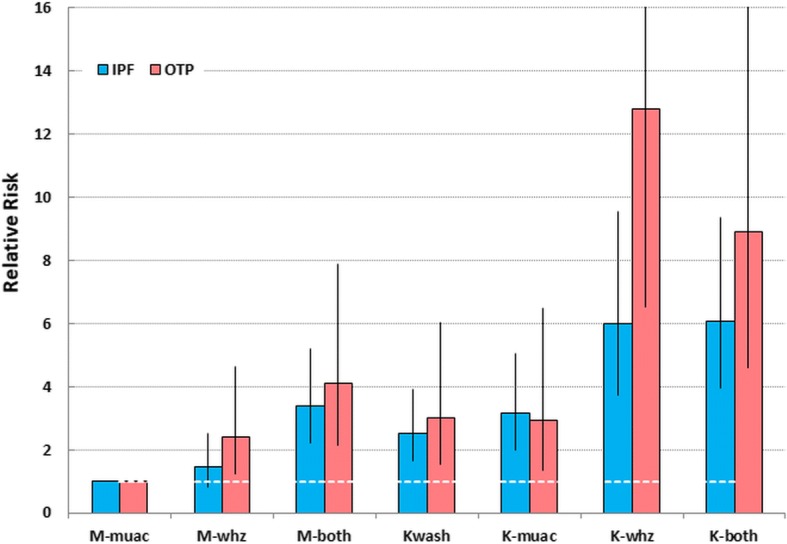
Relative Risks of death (RR) of children with SAM aged 6–60 months by diagnostic criteria and treatment program. The relative risks of death are calculated against marasmic children by MUAC only (M-muac). The error bars are the 95% confidence intervals. *IPF* In-patient Facility; *OTP* Out-patient Treatment Program; *M-muac* MUAC < 115 mm with WHZ ≥ −3Z and no oedema; *M-whz* WHZ < −3Z with MUAC ≥115 mm and no oedema; *M-both* MUAC < 115 mm & WHZ < −3Z and no oedema; *Kwash* nutritional oedema/Kwashiorkor without meeting either MUAC or WHZ criteria; *K-muac* oedema & MUAC < 115 mm with WHZ ≥ −3Z; *K-whz* oedema & WHZ < −3Z with MUAC ≥115 mm; *K-both* oedema & MUAC < 115 mm & WHZ < −3Z; *RR* Relative Risk of death

The children’s countries of residence, mode of treatment and outcomes are shown in Table 1. The corresponding breakdown of the children by diagnostic criteria is given in Additional file 2: Table S2. There were 76,887 children with SAM in the three modes of treatment of which 3588 died. They are divided into the 7 different diagnostic categories of SAM depending upon the criteria present at the time of admission. Their mortality rates are presented by mode of treatment and age group in Table 2. The significances of the paired differences between the diagnostic groups are given Table 3. Figs. 2 and 3 show, respectively, the CFRs and the relative risks of death (RR) calculated against M-muac (the lowest RR) to show how the risks of death for each of the 7 categories of patient relate to one another. The data for the IPF and OTP combined (i.e. excluding those children reclassified as SAM from the SFC) are given in Additional file 3: Table S3.

**Table 1:** Outcome of patients admitted for treatment of SAM by treatment program and country

Data characteristics	Defaulter		Recovery		Death		Non-response		Other		Transfer		Total
	9893	12.9	58,097	75.6	3588	4.7	3490	4.5	1062	1.4	758	1.0	76,887
	n	%	n	%	n	%	n	%	n	%	n	%	n
IPFs by countries													
Angola	34	8.4	346	85.6	24	5.9	0	0.0	0	0.0	0	0.0	404
Burundi	202	13.2	1134	73.9	147	9.6	0	0.0	0	0.0	51	3.3	1534
Chad	59	12.2	364	75.5	26	5.4	0	0.0	0	0.0	33	6.8	482
Congo	21	7.6	236	85.5	19	6.9	0	0.0	0	0.0	0	0.0	276
DRC	249	8.9	2342	83.6	150	5.4	23	0.8	0	0.0	37	1.3	2801
Ethiopia	83	21.1	256	65.1	52	13.2	0	0.0	0	0.0	2	0.5	393
Guinea	18	3.7	417	85.5	44	9.0	0	0.0	0	0.0	9	1.8	488
Kenya	70	15.8	275	62.2	52	11.8	42	9.5	0	0.0	3	0.7	442
Liberia	76	11.3	528	78.5	44	6.5	2	0.3	0	0.0	23	3.4	673
Mali	23	10.4	171	77.4	27	12.2	0	0.0	0	0.0	0	0.0	221
Rwanda	150	9.2	1225	74.7	174	10.6	22	1.3	0	0.0	68	4.1	1639
Sierra Leon	18	16.4	71	64.5	8	7.3	0	0.0	0	0.0	13	11.8	110
Somalia	92	31.6	160	55.0	27	9.3	0	0.0	0	0.0	12	4.1	291
South Sudan	14	8.3	143	84.6	3	1.8	1	0.6	0	0.0	8	4.7	169
Sudan	366	30.3	684	56.7	74	6.1	5	0.4	0	0.0	77	6.4	1206
Tanzania	64	11.4	470	84.1	12	2.1	0	0.0	0	0.0	13	2.3	559
Uganda	550	16.9	2106	64.9	530	16.3	38	1.2	0	0.0	23	0.7	3247
Total	2089	14.0	10,928	73.2	1413	9.5	133	0.9	0	0.0	372	2.5	14,935
OTPs by countries													
Chad	61	29.5	106	51.2	7	3.4	20	9.7	0	0.0	13	6.3	207
DRC	73	2.6	2496	90.2	141	5.1	16	0.6	0	0.0	40	1.4	2766
Ethiopia	17	11.5	91	61.5	23	15.5	17	11.5	0	0.0	0	0.0	148
Kenya	4	8.2	33	67.3	4	8.2	8	16.3	0	0.0	0	0.0	49
Niger	4229	10.5	33,077	82.1	1848	4.6	138	0.3	789	2.0	191	0.5	40,271
South Sudan	76	9.8	665	85.9	20	2.6	12	1.6	1	0.1	0	0.0	774
Uganda	107	9.3	954	83.0	21	1.8	67	5.8	0	0.0	0	0.0	1149
Total	4567	10.1	37,422	82.5	2064	4.5	278	0.6	790	1.7	244	0.5	45,364
SFCs by countries													
DRC	185	7.3	1716	67.6	21	0.8	203	8.0	272	10.7	142	5.6	2539
Kenya	262	22.7	209	18.1	3	0.3	679	58.9	0	0.0	0	0.0	1153
Uganda	2790	21.6	7822	60.7	87	0.7	2197	17.0	0	0.0	0	0.0	12,896
Total	3237	19.5	9747	58.8	111	0.7	3079	18.6	272	1.6	142	0.9	16,588

*IPF* In-patient Facility (Hospital. Therapeutic Feeding Center), *OTP* Out-patient treatment program (Home treatment), *SFC* Supplementary Feeding Centre, *DRC* Democratic Republic of Congo

**Table 2:** Total numbers, deaths, case-fatality rates and relative risk of death of children with SAM by diagnostic criteria, treatment program and age group

SAM type	All patients							In patients (IPF)							Outpatients (OTP)							SFC				
Age group	Total	Dead	*CFR*	RR	95% CI	RR	95% CI	Total	Dead	*CFR*	RR	95% CI	RR	95% CI	Total	Dead	*CFR*	RR	95% CI	RR	95% CI	Total	Dead	*CFR*	RR	95% CI
6–60 m	*#*	*#*	*%*					*#*	*#*	*%*					*#*	*#*	*%*					*#*	*#*	*%*		
M-muac	7191	56	*0.78*	Ref.	─	─	─	759	21	*2.77*	Ref.	─	─	─	780	9	*1.15*	Ref.	─	─	─	5652	26	*0.46*	Ref.	─
M-whz	16,531	332	*2.01*	2.58	1.9–3.4	─	─	674	27	*4.01*	1.45	0.8–2.5	─	─	10,126	280	*2.77*	2.40	0.8–2.5	─	─	5731	25	*0.44*	0.95	0.5–1.6
M-both	40,309	2000	*4.96*	6.37	4.9–8.3	─	─	5985	559	*9.34*	3.38	2.2–5.2	─	─	29,119	1381	*4.74*	4.11	2.2–5.2	─	─	5205	60	*1.15*	2.51	1.6–4.0
Kwash	5882	337	*5.73*	7.36	5.6–9.7	Ref.	─	3735	262	*7.01*	2.54	1.6–3.9	Ref.	─	2147	75	*3.49*	3.03	1.6–3.9	Ref.	─	─	─	─	─	─
K-muac	1669	118	*7.07*	9.08	6.6–12	1.23	1.0–1.5	1138	100	*8.79*	3.18	2.0–5.0	1.25	1.0–1.6	531	18	*3.39*	2.94	2.0–5.0	0.97	0.6–1.6	─	─	─	─	─
K-whz	1088	169	*15.53*	19.95	15–27	2.71	2.3–3.2	465	77	*16.56*	5.98	3.7–9.6	2.36	1.9–3.0	623	92	*14.77*	12.80	3.7–9.6	4.23	3.2–5.6	─	─	─	─	─
K-both	4217	576	*13.66*	17.54	13–23	2.38	2.1–2.7	2179	367	*16.84*	6.09	4–9.4	2.40	2.1–2.8	2038	209	*10.26*	8.89	4–9.4	2.94	2.3–3.8	─	─	─	─	─
Total	76,887	3588	*4.67*					14,935	1413	*9.46*					45,364	2064	*4.55*					16,588	111	*0.67*		
6 - < 18 m	*#*	*#*	*%*					*#*	*#*	*%*					*#*	*#*	*%*					*#*	*#*	*%*		
M-muac	4650	32	*0.69*	Ref.	─	─	─	246	8	3.25	Ref.	─	─	─	298	5	*1.68*	Ref.	─	─	─	4106	19	*0.46*	Ref.	─
M-whz	5906	124	*2.10*	3.05	2.1–4.5	─	─	208	13	6.25	1.92	0.8–4.5	─	─	2901	94	*3.24*	1.93	0.8–4.7	─	─	2797	17	*0.61*	1.31	0.7–2.5
M-both	21,095	1073	*5.09*	7.39	5.2–10	─	─	2703	307	11.37	3.50	1.8–7.0	─	─	14,416	721	*5.00*	2.98	1.2–7.1	─	─	3976	45	*1.13*	2.45	1.4–4.2
Kwash	667	33	*4.95*	7.19	4.5–12	Ref.		290	17	5.86	1.80	0.8–4.1	Ref.		377	16	*4.24*	2.53	0.9–6.8	Ref.		─	─	─	─	─
K-muac	462	20	*4.33*	6.29	3.6–11	0.87	0.5–1.5	231	16	6.93	2.13	0.9–4.9	1.18	0.6–2.3	231	4	*1.73*	1.03	0.3–3.8	0.41	0.1–1.2	─	─	─	─	─
K-whz	194	26	*13.40*	19.47	12–32	2.71	1.7–4.4	63	10	15.87	4.88	2.0–12	2.71	1.3–5.6	131	16	*12.21*	7.28	2.7–19	1.63	0.8–3.2	─	─	─	─	─
K-both	1264	155	*12.26*	17.82	12–26	2.48	1.7–3.6	579	90	15.54	4.78	2.4–9.7	2.65	1.6–4.4	685	65	*9.49*	5.66	2.3–14	2.24	1.3–3.8	─	─	─	─	─
Total	34,238	1463	*4.27*					4320	461	*10.68*					19,039	921	*4.84*					10,879	81	*0.74*		
18 - < 36 m	*#*	*#*	*%*					*#*	*#*	*%*					*#*	*#*	*%*					*#*	*#*	*%*		
M-muac	1973	16	*0.81*	Ref.	─	─	─	242	8	*3.31*	Ref.	─	─	─	376	2	*0.53*	Ref.	─	─	─	1355	6	*0.44*	Ref.	─
M-whz	8044	171	*2.13*	2.62	1.6–4.4	─	─	242	7	*2.89*	0.88	0.3–2.4	─	─	6143	158	*2.57*	4.84	1.2–19	─	─	1659	6	*0.36*	0.82	0.3–2.5
M-both	16,506	774	*4.69*	5.78	3.5–9.5	─	─	2110	169	*8.01*	2.42	1.2–4.9	─	─	13,289	594	*4.47*	8.40	2.1–34	─	─	1107	11	*0.99*	2.24	0.8–6.0
Kwash	2510	138	*5.50*	6.78	4.1–11	Ref.		1393	99	*7.11*	2.15	1.1–4.4	Ref.		1117	39	*3.49*	6.56	1.6–27	Ref.		─	─	─	─	─
K-muac	655	43	*6.56*	8.10	4.6–14	1.19	0.8–1.7	444	35	*7.88*	2.38	1.1–5.1	1.11	0.8–1.6	211	8	*3.79*	7.13	1.5–33	1.09	0.5–2.3	─	─	─	─	─
K-whz	574	83	*14.46*	17.83	11–30	2.63	2.0–3.4	173	32	*18.50*	5.60	2.6–12	2.60	1.8–3.7	401	51	*12.72*	23.91	5.9–97	3.64	2.4–5.4	─	─	─	─	─
K-both	2139	275	*12.86*	15.85	9.6–26	2.34	1.9–2.8	976	160	*16.39*	4.96	2.5–9.9	2.31	1.8–2.9	1163	115	*9.89*	18.59	4.6–75	2.83	2.0–4.0	─	─	─	─	─
Total	32,401	1500	*4.63*					5580	510	*9.14*					22,700	967	*4.26*					4121	23	*0.56*		
36–60 m	*#*	*#*	*%*					*#*	*#*	*%*					*#*	*#*	*%*					*#*	*#*	*%*		
M-muac	568	8	*1.41*	Ref.	─	─	─	271	5	*1.85*	Ref.	─	─	─	106	2	*1.89*	Ref.	─	─	─	191	1	*0.52*	Ref.	─
M-whz	2581	37	*1.43*	1.02	0.5–2.2	─	─	224	7	*3.13*	1.69	0.5–5.3	─	─	1082	28	*2.59*	1.37	0.3–5.7	─	─	1275	2	*0.16*	0.30	0.0–3.3
M-both	2708	153	*5.65*	4.01	2.0–8.1	─	─	1172	83	*7.08*	3.84	1.6–9.4	─	─	1414	66	*4.67*	2.47	0.6–10	─	─	122	4	*3.28*	6.26	0.7–55
Kwash	2705	166	*6.14*	4.36	2.2–8.8	Ref.	─	2052	146	*7.12*	3.86	1.6–9.3	Ref.	─	653	20	*3.06*	1.62	0.4–6.8	Ref.	─	─	─	─	─	─
K-muac	552	55	*9.96*	7.07	3.4–15	1.62	1.2–2.2	463	49	*10.58*	5.74	2.3–14	1.49	1.2–2.0	89	6	*6.74*	3.57	0.7–17	2.20	0.9–5.3	─	─	─	─	─
K-whz	320	60	*18.75*	13.31	6.4–27	3.06	2.3–4.0	229	35	*15.28*	8.28	3.3–21	2.15	1.5–3.0	91	25	*27.47*	14.56	3.5–60	8.97	5.2–15	─	─	─	─	─
K-both	814	146	*17.94*	12.73	6.3–26	2.92	2.4–3.6	624	117	*18.75*	10.16	4.2–25	2.64	2.1–3.3	190	29	*15.26*	8.09	2.0–33	4.98	2.9–8.6	─	─	─	─	─
Total	10,248	625	*6.10*					5035	442	*8.78*					3625	176	*4.86*					1588	7	*0.44*		

*IPF* In-patient Facility (Hospital. Therapeutic Feeding Center); *OTP* Out-patient treatment program (Home treatment); *SFC* Supplementary Feeding Center; *M-muac* MUAC < 115 mm with WHZ ≥ −3Z and no oedema (marasmus by MUAC only); *M-whz* WHZ < −3Z with MUAC ≥115 mm and no oedema (marasmus by WHZ only); *M-Both* MUAC < 115 mm & WHZ < −3Z and no oedema (marasmus by both diagnostic criteria); *Kwash* nutritional oedema/Kwashiorkor without meeting either MUAC or WHZ criteria; *K-muac* oedema & MUAC < 115 mm with WHZ ≥ −3Z (marasmic kwashiorkor by MUAC only); *K-whz* oedema & WHZ < −3Z with MUAC ≥115 mm (marasmic kwashiorkor by WHZ only); *K-Both* oedema & MUAC < 115 mm & WHZ < −3Z (marasmic kwashiorkor by both diagnostic criteria); *CFR* Case Fatality Rate %; *RR* Relative Risk of death; *95% CI* 95% Confidence interval. Significance tests are given in Table 3

**Table 3:** Significance levels of comparisons between diagnostic groups using Marascuilo post-hoc analysis procedure

Children with marasmus				
Comparisons	All	IPF	OTP	SFC
0–60 m	p	p	p	p
muac v whz	0.000	ns	0.001	ns
muac v both	0.000	0.000	0.000	0.000
whz v both	0.000	0.000	0.000	0.000
6 - < 18 m				
muac v whz	0.000	ns	ns	ns
muac v both	0.000	0.000	0.000	0.003
whz v both	0.000	0.017	0.000	ns
18 - < 36 m				
muac v whz	0.000	ns	0.000	ns
muac v both	0.000	0.001	0.000	ns
whz v both	0.000	0.000	0.000	ns
36–60 m				
muac v whz	ns	ns	ns	ns
muac v both	0.000	0.000	ns	ns
whz v both	0.000	0.017	0.019	ns
Children with kwashiorKor/marasmic-kwashiorKor				
Comparisons	All	IPF	OTP	
0–60 m	*p*	*p*	*p*	
K-muac v Kwash	ns	ns	ns	
K-whz v Kwash	0.000	0.000	0.000	
K-both v Kwash	0.000	0.000	0.000	
K-muac v K-whz	0.000	0.001	0.000	
K-muac v K-both	0.000	0.000	0.000	
K-whz v K-both	ns	ns	0.041	
6 - < 18 m				
K-muac v Kwash	ns	ns	ns	
K-whz v Kwash	0.014	ns	ns	
K-both v Kwash	0.000	0.000	ns	
K-muac v K-whz	0.007	ns	0.006	
K-muac v K-both	0.000	0.002	0.000	
K-whz v K-both	ns	ns	ns	
18 - < 36 m				
K-muac v Kwash	ns	ns	ns	
K-whz v Kwash	0.000	0.003	0.000	
K-both v Kwash	0.000	0.000	0.000	
K-muac v K-whz	0.000	0.012	0.001	
K-muac v K-both	0.000	0.000	0.002	
K-whz v K-both	ns	ns	ns	
36–60 m				
K-muac v Kwash	0.047	ns	ns	
K-whz v Kwash	0.000	0.011	0.000	
K-both v Kwash	0.000	0.000	0.000	
K-muac v K-whz	0.007	ns	0.002	
K-muac v K-both	0.000	0.002	ns	
K-whz v K-both	ns	ns	ns	

Abbreviations are given in Table 2; *ns* not significant at *p* < 0.05

Considering marasmus, overall the mortality is significantly higher in those with WHZ < −3Z than in those with MUAC < 115 mm. WHZ-only children also had a higher mortality in each of the age groups although this does not reach significance in the children 36 to 60 months. The children who had both anthropometric deficits had more than twice the mortality of either the WHZ-only or the MUAC-only groups. The children with complicated SAM (IPF) and those without complications (OTP) show the same pattern of mortality, but, as expected, it is higher in the complicated than uncomplicated cases. For both the complicated and un-complicated cases examined separately, the higher mortality with WHZ than with MUAC was present in each of the age groups. There was no indication that MUAC mortality dominated death in either the younger or older age groups. WHZ-only consistently had equivalent or higher mortality than MUAC-only across all age groups.

For the oedematous children, those without severe wasting (Kwash) had about the same mortality rate as the marasmic children with both anthropometric deficits (M-both), but higher than children with single deficits. It was significantly higher in the complicated than the uncomplicated cases; this is presumably because only children with mild or moderate oedema are admitted to the OTPs whereas those with severe oedema are always admitted to the IPF as well as those with complications. Table 2 also shows the RR of oedematous children calculated against those without an anthropometrical deficit (Kwash). The children with oedema and a low MUAC (K-muac) did not have a higher mortality than oedematous cases without a low MUAC (Kwash) in either the OTP or IPF so that the addition of the MUAC criterion to oedematous cases did not increase their mortality risk (K-muac). This is in marked contrast to the children with oedema and a WHZ below <−3Z (K-whz); these particular children in both the OTP and IPF experienced a very high CFR and a RR of between 2 and 3 times the risk for children with either Kwash or K-muac. Their mortality was, well above the Sphere standards. The fact that the severely oedematous cases (+++) were not included in the OTP group did not seem to ameliorate the mortality rate of the K-whz children treated as outpatients compared to those treated in the IPFs. Furthermore, addition of a low MUAC to those who already had a low WHZ and oedema (K-both) did not further augment the mortality rate over those with oedema and a low WHZ (K-whz). Thus, the presence or absence of a MUAC below 115 mm in the oedematous children appears to be without significance in terms of increasing their mortality risk; this is in marked contrast to WHZ where there was a profound increase in risk.

In comparison with those children with only a MUAC < 115 mm, each diagnostic category had a significantly higher relative CFR and RR (Figs. 2 & 3). In the case of the children with a WHZ < −3Z and oedema the death rate was between 6 to 12 times as high as those with only a MUAC < 115 mm. These observations did not change by age group, although the numbers of deaths in the older age group and in the children admitted to SFC was insufficient to reach significance.

### Meta-analysis showing regional differences

**Fig. 4:**
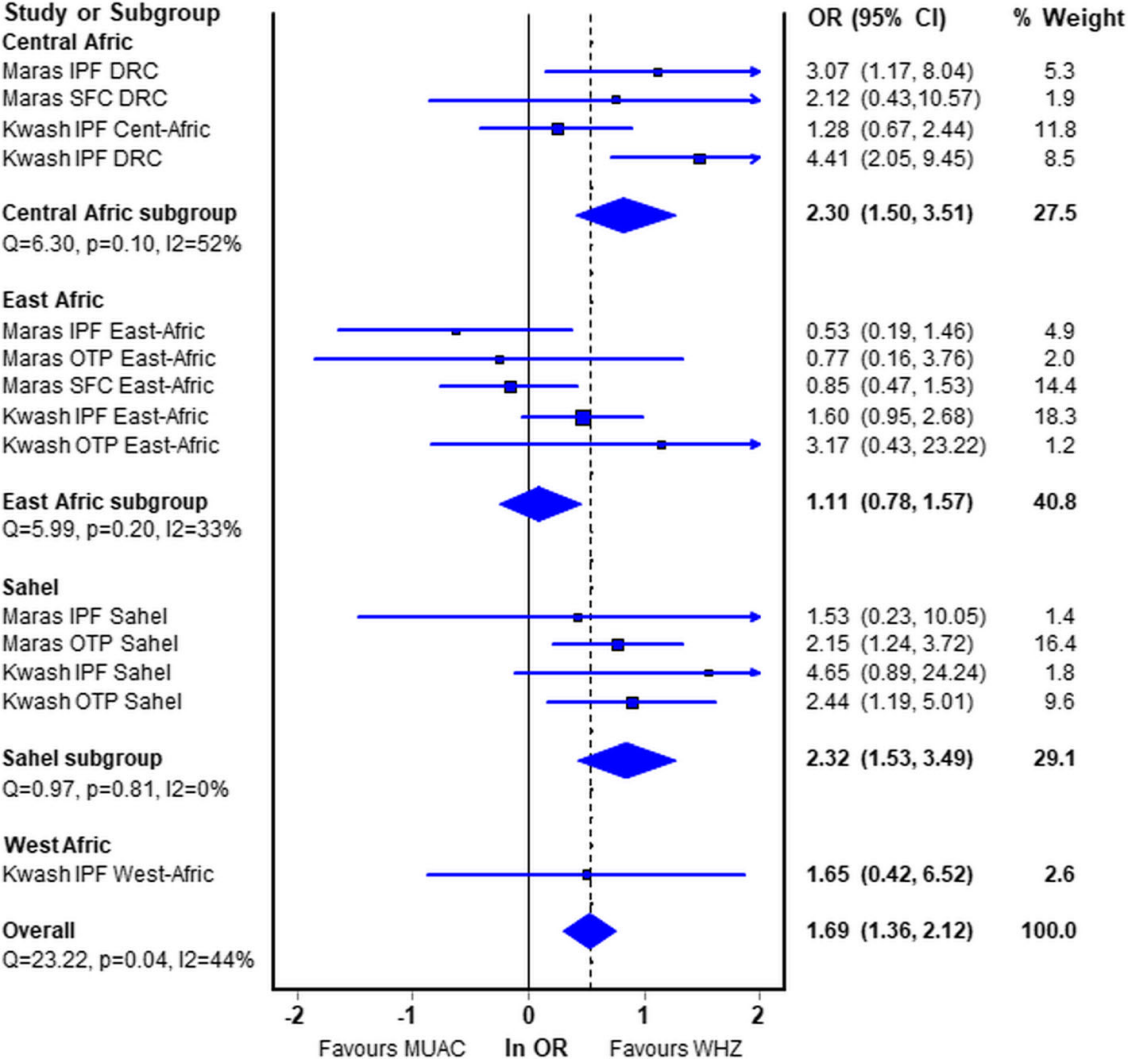
Forest plot by region comparing Odds ratios of the risk of death of M-muac v M-whz. *Maras* Marasmus (M-muac vs M-whz); *Kwash* nutritional oedema (K-muac vs K-whz); *IPF* In-patient Facility; *OTP* Out-patient Treatment Program; *SFC* Supplementary Feeding Center; *DRC* Democratic Republic of Congo. The countries contributing data from each region are given in Additional file 2: Table S3. The statistical data are given in Additional file 4: Table S4 and the sensitivity analysis in Additional file 5: Table S5. Please note that the data for OTP West Africa has been omitted from this plot due to issues of presentation, but the data including this program is given in the additional files. In each of the forest plots “favours WHZ” indicates that the Odds ratio for death is higher in children with WHZ < −3Z than with a MUAC of < 115 mm; “favours MUAC” indicates that the Odds ratio for children with a MUAC < 115 mm is higher than those with WHZ < −3Z

Figure 4 shows the forest-plot of the meta-analysis by programs from the different regions of Africa comparing WHZ-only with MUAC-only. The countries that constituted each of the regions are given in Additional file 4: Table S4 (OTP of oedematous children from West Africa is omitted from the plot for formatting reasons. The odds ratio in favour of K-whz over K-muac was very high – 17.8, CI = 7.5–41.9 – but is included in the statistics and sensitivity analyses given in Additional file 5: Table S5). Overall the odds ratio of death was 1.7 times higher for the children with only a WHZ < −3Z that those with only a MUAC < 115 mm. There were regional differences; for each of the modes of treatment (IPF, OTP, SFC) WHZ carried a higher risk of death than MUAC in the Central, West and Sahelian countries for both non-oedematous and oedematous children. In contrast, marasmic children in the East African group (Kenya, Tanzania, Uganda, Ethiopia) had a lower risk of death with WHZ than with MUAC, albeit not significantly.

**Fig. 5:**
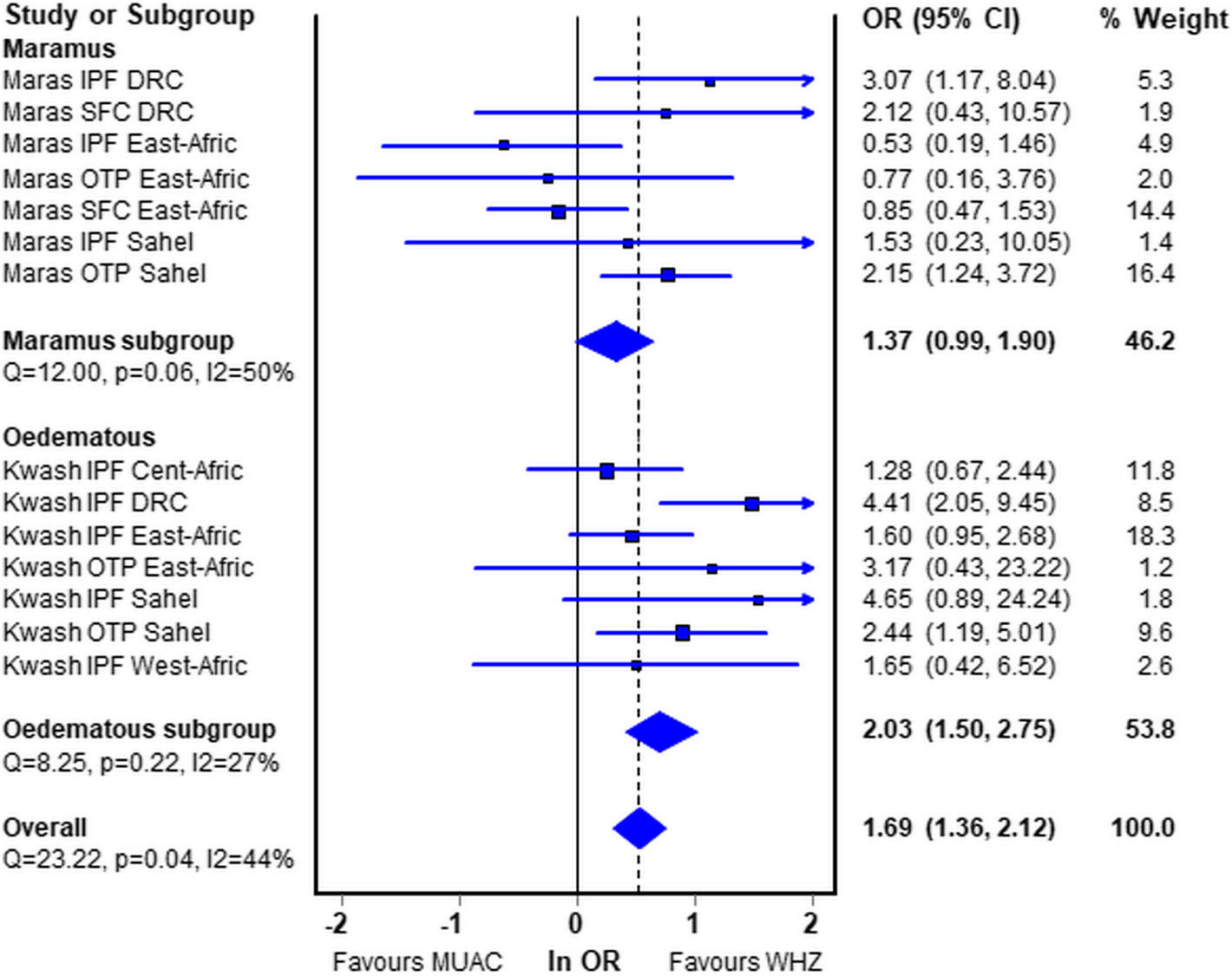
Forest plot by oedema status (marasmus vs kwasiorkor) region comparing Odds ratios of the risk of death of children admitted with WHZ < −3Z only against MUAC < 115 mm only. *Maras* Marasmus (M-muac vs M-whz); *Kwash* nutritional oedema (K-muac vs K-whz); *IPF* In-patient Facility; *OTP* Out-patient Treatment Program; *SFC* Supplementary Feeding Center; *DRC* Democratic Republic of Congo

The same data are analysed by oedema status and presented in Fig. 5. They show that for marasmic children the odds ratio is marginally significant at 1.37 (95% CI, 0.99–1.90) whereas for oedematous children the odds ratio for death with K-whz is twice that of K-muac (2.03, CI 1.50–2.75). The East African data in particular shows a discordance between the children without oedema and those with oedema.

### Simpson’s paradox

In Table 4 the case fatality rates for the patients with MUAC-alone, WHZ-alone and both combined are shown and compared with all the cases with a low MUAC (All-muac = M-muac + M-both) and all the WHZ cases (All-whz = M-whz + M-both). As shown in Table 2, for marasmic cases each comparison is highly significant with WHZ having a higher mortality than MUAC when the children fulfilling each criterion alone are considered. Table 4 shows that when the children with both defects are added to the WHZ and the MUAC categories not only is the difference now non-significant, but the CFR is *reversed* so that MUAC now appears to have a *higher* mortality than WHZ. For the oedematous children the ratio is not quite reversed, but the apparent mortality of MUAC has increased and that of WHZ decreased. When all the SAM children are considered, that is oedematous and non-oedematous SAM combined, again the relative mortality is significantly higher in children with a low WHZ when considered alone, but this is reversed when the children with both criteria are incorporated into the MUAC and the WHZ groups.

**Table 4:** Effect of combining the diagnostic groups together to show Simpson’s paradox

Effect of combining diagnostic groups					
Subgroups	Total	dead	CFR	comparisons	*p*
	#	#	%		
All-muac v All-whz without oedeam					
M-muac	7191	56	0.78	muac v whz	0.000
M-whz	16,530	332	2.01	muac v both	0.000
M-both	40,307	2000	4.96	whz v both	0.000
				Cramer’s V	0.044
M-All-muac	47,498	2056	4.33	χ^2^ = 3.01	0.083
M-All-whz	56,837	2332	4.10	Cramer’s V	0.005
All-muac v All-whz with oedema					
K-muac	1669	118	7.07	muac v whz	0.000
K-whz	1088	169	15.53	muac v both	0.000
K-both	4217	576	13.66	whz v both	0.411
				Cramer’s V	0.115
K-All-muac	5886	694	11.79	χ^2^ = 9.75	0.002
K-All-whz	5305	745	14.04	Cramer’s V	0.028
All-muac v All-whz with and without oedema					
M + K-muac	8860	174	1.96	muac v whz	0.000
M + K-whz	17,618	501	2.84	muac v both	0.000
M + K-both	44,524	2576	5.79	whz v both	0.000
				Cramer’s V	0.025
M + K - All-muac	53,384	2750	5.15	χ^2^ = 2.16	0.141
M + K - All-whz	62,142	3077	4.95	Cramer’s V	0.004

The effect of grouping M-both and K-both with the single deficits to produce erroneous results due to mathematical coupling. *All-muac* is defined as M-muac + M-both or K-muac + K-both; *All-whz* is defined as M-whz + M-both or K-whz + K-both; *Cramer’s V* (a measure of the degree to which the two categories are associated, 0 = no association, 1 = identity) calculated for MUAC v WHZ only

This is an example of extreme confounding, in this case due to mathematical coupling, leading to Simpson’s paradox where there is a paradoxical reversal of the estimated mortality risk to give an erroneous result when groups of children are inappropriately combined.

## Discussion

To judge whether using any of the 3 recognised WHO diagnostic criteria for SAM can be dropped, the critical factor is to focus on the potential fate of those children who would then become systematically ineligible for treatment and omitted from care. About 45% of SAM children in the community fulfil the WHZ but not the MUAC criterion [42]. Any advocates that propose elimination of children fulfilling WHZ criteria from treatment should demonstrate that both their risk of death and the other detrimental effects of being severely malnourished are trivial or at least substantially lower than those diagnosed using MUAC. Our data demonstrate that children with a WHZ less than -3Z but a MUAC of above 115 mm are at high risk of death, at least as high as or higher than those with a low MUAC across each of the age groups. On this evidence there is no place to cease the use of WHZ as an independent criterion for the admission and treatment of SAM children; agencies and governments that have adopted a MUAC-only policy should reflect upon the provisions of their guidelines, and where appropriate reverse the MUAC-only policy and maintain using the current WHO guidance.

It would appear that the contentions put forward by Briend et al. and others [43, 46, 49–51] are incorrect (see also the companion paper [60] where the literature is reviewed). In particular the contention that children with a WHZ below -3Z are relatively healthy with a low risk of death. This is justified by reference to a review on leg length and beauty which does not mention wasting let alone marasmus [64]. This contention is without evidence and contrary to common sense and clinical experience in all age groups [65]. In fact there are abundant data to confirm that low WHZ itself caries a substantial risk of death [66–78]. Although none of these papers also measured MUAC to determine whether the deaths occurred in patients that had a concomitant low MUAC and would therefore be identified by both criteria. Given the low rate of concordance it is unlikely that the majority of deaths occurred in children with both deficits. Of interest is the paper by Katz et al. [72] who show a much higher mortality risk for WHZ (< 80% NCHS) in older than younger children, when they are less likely to have a low MUAC. Briend is a co-author on O’Neill et al’s paper [76] where BMI-for-age (closely related to WHZ) is a better predictor of mortality than MUAC and WHZ itself has a dramatic impact on mortality.

Briend et al. also assert that the discrepancy is simply because children with a low WHZ have longer legs, whereas the only papers that have addressed this issue show this is, at best, a minor contributor [32, 42, 79] and the original authors are clear that long legs do not explain the discrepancy between WHZ and MUAC. Long legs do not account for the fact that in most surveys different children are identified by WHZ and MUAC. The discrepancy is more likely due to differences in body build, rather than linear growth; a concern of auxologists in early studies which has not been considered in deriving modern standards [9, 80]. What is not clear is whether endomorphic children, with narrow torsos, who are more likely to have a lower WHZ, have a different risk of death than exomorphic children. We speculate that endomorphic children have a higher risk of death than exomorphic children, in the face of privation, as their body fat and muscle mass is relatively low; however, there are no data to support or refute such an hypothesis.

Briend et al. also dispute that the deficits are additive or that the two diagnostic criteria are complementary. Our data also show that this assertion to be false. Those with both deficits had over twice the mortality of those with a MUAC< 115 mm alone. Indeed, some of the data suggests that the deficits might by synergistic. Even WHZ and stunting (height-for-age) combined show an additive effect [74]. Although stunting has not been taken into account in our analysis it is another confounder for all prognostic assessments of SAM. In our companion paper [60] we present a literature review of studies comparing WHZ and MUAC to predict death of malnourished children; the conclusions reached are in broad agreement with the present study.

### Confounding and Simpson’s paradox

Simpson’s paradox is an example of extreme confounding where the actual results of a comparison can be reversed resulting in the less important variable becoming the dominant variable. This can occur with all analyses of categorical data, including simple Chi-squared analyses, logistic regression and ROC curve analyses. When categorical and continuous/ordinal data are combined the same phenomena can also occur and is then termed Lord’s paradox and when both sets of data are continuous “suppression effects”. They all have the same basis and are due to inadequate categorisation of subjects, confounding, mathematical coupling, inappropriate adjustment and unmeasured effects [81]. Even if the results are not actually reversed, mathematical coupling and the other confounders give erroneous results. The classical examples compare surgical operations for renal stones, psychiatric hospital admissions and death from diabetes [82] where those with more severe disease are not analysed separately from those with milder forms of a condition. Combining patients inappropriately led to erroneous statics and conclusions in each case. In the case of SAM, the same phenomenon occurs when children with both deficits, who have a higher mortality risk, are combined with those with single deficits who have a lower mortality risk, particularly when WHZ children are at higher risk of death than those with a low MUAC. Thus, inappropriate categorisation of patients, the presence of confounding or data from patients that are included in both arms of a comparison, even if the results are not completely reversed the magnitude of the difference in mortality can be grossly in error. Stochastic studies that relate subsequent events to antecedent parameters (such as subsequent death to antecedent anthropometry or adult blood pressure to birth weight etc. [83]) are particularly liable to error by confounding, sometimes to the extent of paradoxical reversal.

Consider Table 5. Here we present comparison of two criteria X and Y, with different numbers of subjects and deaths (the table can be reproduced in a spreadsheet to examine the paradox). In scenario A, there are no deaths at all in children with X alone, but when combined with children with both X and Y, the two deficits appear to have exactly the same mortality risk. In scenario B there is a lower CFR with X than with Y, but when those with both deficits are added the apparent CFR is reversed. In scenario C the two deficits have the same morality risk, but when combined with those with both deficits X appears to have a higher mortality. The percentage of total deaths identified using criterion X is much lower than with Y in each scenario even though the CFR with X appears to be higher when those with both groups are incorporated. In this case the error is due to mathematical coupling.

**Table 5:** The effect of group combination on the proportion of deaths identified by X or Y criteria

	Total	Dead	CFR	CFR	X deaths	Y deaths
	#	#	%	X/Y	%	%
Scenario A						
*X*	500	0	0.0			
*Y*	1500	30	2.0	0.0		
*Both X & Y*	500	30	6.0			
*Total*	2500	60	2.4			
*All-X*	*1000*	*30*	3.0		50%	
*All- Y*	*2000*	*60*	3.0	1.0		100%
Scenario B						
*X*	500	6	1.2			
*Y*	1500	30	2.0	0.6		
*Both X & Y*	500	30	6.0			
*Total*	2500	66	2.6			
*All- X*	*1000*	*36*	3.6		55%	
*All- Y*	*2000*	*60*	3.0	1.2		91%
Scenario C						
*X*	500	10	2.0			
*Y*	1500	30	2.0	1.0		
*Both X & Y*	500	30	6.0			
*Total*	2500	70	2.8			
*All- X*	*1000*	*40*	4.0		57%	
*All- Y*	*2000*	*60*	3.0	1.3		86%

In Scenario A, X does not have any mortality by itself, but when the subjects with both criteria are included X and Y appear to have the same mortality rate. Using only criterion X would select those children with zero mortality and those with both X and Y criteria and miss all the deaths related to criterion Y

In Scenario B, there is a lower mortality with criterion X, however when the subjects fulfilling both criteria are included the relative case fatality rates are reversed so it appears now that X is a superior diagnostic parameter than Y. Yet its use only identifies 55% of the deaths

In Scenario C, both X and Y have the same mortality rates but when the subjects with both criteria are included Y appears to be a superior diagnostic criterion. Yet this only leads to identification of 57% of deaths

The columns % deaths shows the percentage of all deaths that would occur in children with criterion X or criterion Y as the single diagnostic tool. Criterion Y identifies more deaths than criterion X, but when the children with both criteria are included criterion X appears to have a higher case fatality rate

Mathematical coupling occurs where “*one variable directly or indirectly contains the whole or part of another, and the two variables are analysed using standard statistical techniques*” [84, 85]. This nearly always results in erroneous results and appears to be the case in all the papers where children with M-both or K-both have been incorporated into the data analysed (see companion paper II [60]) and is the case with our patient’s data (Table 4). It is for this reason that we have not used the children with both deficits in the meta-analysis and separated them in Table 2. Other types of confounding can also cause errors and even generate Simpson’s paradox. Some are know; for example, the meta-analysis showed that MUAC had a higher risk of death for marasmic children in three different programs in East Africa and the combined risk is marginally in favour of WHZ, but when the children with oedema are added to the them Fig. 5 shows that the risk is changed so that overall WHZ has a significantly higher mortality. Some confounders have not been recorded in our data such as HIV, diarrhoea and family circumstances; and some are unknown such as birth weight. The use of such data to guide policy must be circumspect and confirmed. These problems are usually described in regression or correlation analyses, but the phenomenon also applies to logistic regression and ROC curve analysis.

### ROC curves

Comparison of WHZ and MUAC related all-cause mortality using ROC curve comparisons generally show that the area under the curve is greater with MUAC than WHZ and is the only reason why MUAC is considered to be a “superior” prognostic of death than WHZ [51, 68, 86–88]. The question arises as to why these ROC curves have provided the opposite results to the findings in the present analysis. We have discussed many problems of ROC curve interpretation in relation to MUAC and WHZ assessment of future all-cause mortality elsewhere [52]. In particular, if they identify mostly or completely different children at high risk of death we argue that they are complementary and are not competing to identify the same child deaths. Thus, even if one diagnostic has a higher mortality risk than the other, if different deaths are identified, they are both useful prognostic indicators. If the objective of treatment is to try to prevent all the deaths and not only deaths which are related to one or the other diagnostic criterion both diagnostic criteria must be used. This is despite the fact that none of the anthropometric criteria are very good prognostic indicators and the differences between them are marginal. Each relates poorly to clinical or physiological abnormalities [89–92].

There has been a debate in the statistical literature about the problems of bias in ROC curves [93] which are particularly relevant to prognostic models of stochastic data (i.e. a future event) [94–96]. There have been attempts to combine time-to-event analysis with ROC curve analysis [97] and also comparison of crude data against smoothed data analysis with small sample sizes of individuals [98], but frequently there are anomalous results [99]. It is noteworthy that most of the papers presenting ROC curves of WHZ and MUAC have not given the confidence intervals of the curves [100].

The ROC curves that have been published in support of MUAC being “superior” to WHZ are all subject to Simpson’s paradox because the children with both defects have not been analysed separately. They are also confounded due to the presence of oedema, HIV, convulsions, measles and other biases that affect children with a low MUAC and WHZ differently. As Tu et al. [81] state: “*Incorrect use of statistical models might produce consistent, replicable, yet erroneous results*”. We contend that this stricture applies to each of the reports using ROC curve analysis and the conclusions based upon these data are consistently erroneous. When we separate those children with both defects from those with either one or the other we reach quite the opposite conclusion; WHZ < −3Z has a higher mortality risk than MUAC < 115 mm.

When the WHZ ROC curve has a greater area under the curve than the MUAC curve, the data are not presented as the authors consider there has been a mistake [101]. When sensitivity is compared there are some situations where WHZ out performs MUAC [39]; this is uncommon with combined data but is usually ignored. Although papers, with completely inadequate data are erroneous and have been heavily criticised [102], they are still being quoted to justify a MUAC-only policy [103].

### Marasmic-kwashiorkor

The finding of an exceptionally high mortality among children with oedema and a WHZ < −3Z, but no augmentation of mortality in those with oedema and a MUAC < 115 mm, is an unexpected new finding which to our knowledge has not been previously reported. The only explanation we can think of is that the weight of the oedema fluid will increase the WHZ so that the oedema-free WHZ may be much less than -3Z. However, the fact that the high mortality occurred in the OTP, with mild to moderate oedema, as well as the IPF with more severe oedema is against this as a complete explanation. The severity of the oedema does not appear to substantially affect the increased mortality risk when combined with a WHZ < −3Z. It appears that there is a qualitative difference between oedematous SAM related to a low WHZ and a low MUAC. The effect is seen in both younger and older children, so that age is not a satisfactory explanatory factor. Nevertheless, this observation may explain a controversy surrounding the relative increase in risk of children with marasmic-kwashiorkor over those with either marasmus or kwashiorkor alone. The WHO guidelines state that children who have been selected by screening children with MUAC and have mild or moderate oedema can be safely treated as outpatients [104], whereas clinicians treating children admitted using WHZ criteria maintain that children who also have oedema are at very high risk of death. Our data may reconcile this difference as the difference appears to depend upon whether a child has been diagnosed using the WHZ or the MUAC criterion. It should be noted that the high mortality in our data for oedema plus WHZ < −3Z was for both children with a MUAC of more and less than 115 mm. As the two anthropometric parameters appear to identify different risks of death, we suggest that all oedematous children should have their WHZ assessed and if they have marasmic-kwashiorkor with a WHZ of less than -3Z they should be treated as in-patients, whereas if their only anthropometric deficit is a low MUAC they can continue to be treated as out-patients.

### Limitations of the study

Although there were a very large number of children’s data amassed for this analysis, when those with “both” criteria were omitted from the analyses there were few deaths in some of the categories of interest, making it necessary to combine data from different sites. This could have resulted in a “clustering” effect. However, the large number of sites contributing data should have ameliorated such effects.

The percent of children with both WHZ and MUAC criteria without oedema in the IPF, OTP and SFC is 81, 73 and 31% respectively; for those with oedema the overlap was 58 and 64%. Compared to the overlap of SAM children identified in nutritional surveys (16.5%) in the community there is an excess of children fulfilling both criteria. There is a clear ascertainment bias as well as potential co-morbidity and stochastic biases [52]. The increase is numbers of children with both criteria may have contributed to the appearance of Simpson’s paradox. The increase in the degree of overlap going from the least to the most intensive management reflects the severity of the cases being admitted as well as the diagnostic procedures and policy of the institutions/agencies involved. As a child deteriorates s/he is more likely to be complicated, to fulfil more than one diagnostic category and to be admitted for more intensive treatment. The ascertainment bias indicates that the data do not reflect the children with SAM in the community and disproportionally describes the experience of more severely affected children than are generally found during a community survey. Nevertheless, by dividing the children into the 7 different diagnostic categories, and omitting those satisfying both criteria from comparison of MUAC and WHZ CFRs, we consider that this bias has been ameliorated; thus, the M-muac and M-whz children included are more likely to represent the M-muac and M-whz children found in the community than the M-both children. Ascertainment bias is likely to be a more important consideration in studies that fail to separate the categories of patients and also those that include oedematous children in the analysis.

The fact that similar results were obtained in each mode of treatment supports the conclusion that children with a low WHZ have a higher mortality risk than those with a low MUAC, in all age groups and modes of treatment irrespective of the criterion used to admit the child for treatment initially. The fact that similar results were obtained with the three modes of treatment, each with a different degree of overlap, indicates that this is not a primary cause for the paradoxical results found.

We did not have data for other potential confounding influences on our analyses; in particular variables such as infection rates (TB, HIV, malaria etc.), socio-economic status, birth weight etc. all of which could confound studies of this nature. Furthermore, the causes of death were not clear, but were presumed to be related to their severe acute malnutrition.

There were a considerable numbers of defaulters from all the programs (Table 1). We included them in our study because they were at risk of death up to the time they defaulted and defaulting generally occurs after most of the deaths due to SAM have taken place [56]. However, this applies mainly to the IPF patients were it is know for certain whether a child has died or not prior to default. The patients in OTP and SFC are at home and attend the program sites weekly or less often; they are declared defaulters if they do not attend for two consecutive visits. As home visits are rarely performed, a proportion of these “defaulters” will have died; thus, the OTP and SFC’s mortality rates should be regarded as minimum mortality rates and not actual mortality rates. For this reason defaulting is a potential major bias particularly for the OTP and SFC data. Table 6 shows the percent of defaulting by category and treatment mode. There was no difference in the rate of defaulting for children with M or K muac and whz in the IPF. However, in the OTP and SFC the difference was significant; children with M-muac had a higher percent of defaults than those with M-whz. If the same proportion of defaulting children died with each criterion, then there would be more deaths added to the M-muac group which would increase their CFR. With the oedematous children in the OTP the defaulting rate difference was the opposite so that this would increase the oedematous children’s K-whz CFR more than the K-muac rate. For these reasons the OTP and SFC data are less reliable than the IPF data. This criticism applies to all studies of patients attending OTP programs unless there is universal home-visiting follow-up to ascertain the reason for the patient not attending the OTP/SFC site. This is very rarely done. One study from Niger (Médecins Sans Frontières personal communication) indicated that about 10% of “defaults” could be reclassified as deaths, but this figure is likely to be context specific, so we have not assumed any particular “correction” factor for our analyses.

**Table 6:** Percent of patients who defaulted by diagnostic category and treatment

	IPF		OTP		SFC	
Category	%	χ2 P Cramer’s V	%	χ2 P Cramer’s V	%	χ2 P Cramer’s V
M-muac	10.5	0.015	7.4	10.6	22.3	29.4
M-whz	10.8	*0.901*	4.7	*0.001*	17.2	*0.000*
		0.003		0.031		0.048
K-muac	12.6	1.735	4.5	6.953		
K-whz	15.7	*0.188*	8.7	*0.008*		
		0.032		0.076		
M-both	17.1		12.3		18.5	
K-both	14.1		13.8			

Abbreviations are given in Table 2

Oedema is rarely seen during a nutritional survey because the time course of kwashiorkor is brief relative to marasmus; thus, even if the prevalence is low during a survey, the incidence can be considerable; this is evident from the high proportion of oedematous cases in some of the IPF studies. The ratios of oedematous SAM to non-oedematous SAM in treatment facilities always greatly exceed those in surveys.

In order to choose an appropriate admission policy that avoids death due to SAM, children should be categorised in the analysis into those with each criterion alone or both separately and those who have oedema or death from diseases not related to their nutritional status should be analysed separately.

## Conclusions

Some within the nutritional community has been deceived by replicated ROC analyses because of mathematical coupling and confounding which may even lead to Simpson’s paradox. Children with a low weight-for-height are at substantial risk of death at least as great as those with a low MUAC. But, because the two parameters identify different children they cannot be fairly compared as diagnostic markers for the *same* risks so that the comparison of areas under ROC curves, even if this were statistically a legitimate comparison, is largely meaningless. In studies of cancer, for example, one would not compare the ROC curves of all-cause mortality in patients with bladder and renal cancer and decide, because one curve was “superior” to drop attempts to diagnose the other condition.

MUAC-only programs will fail to identify up to 45% of SAM children at high risk of death without the possibility of their being diagnosed or treated, and this omission will fail to be recognised by “coverage surveys” using MUAC screening alone. In our opinion MUAC-only programs are unethical wherever it is possible to also measure WHZ because they contravene the dictates of Hippocrates. In emergency situations where health services are overwhelmed MUAC- only programs could be justified at the outset of the emergency; however, in emergencies older children have a proportionately higher increase in prevalence of SAM (unpublished) than younger children and emergency interventions should not neglect this group of children. The research priority should be to develop innovative ways of identifying those children with a low WHZ in community screening programs. Stereo-photography has been used for many years [105] but with modern technology this has become practical [106–109]. Such data may also solve the problems of body build in determination of the difference between those with a low WHZ and a low MUAC and explore the relative risks of endomorphic and exomorphic children. In the meantime WHZ should continue to be used in all health facilities to identify and treat all those children with SAM by WHZ only.

Our data also suggest that those children with oedema and a low WHZ, but not those with a low MUAC, are at very high risk of death and should be referred to an IPF for initial treatment; they should form a separate diagnostic category and considered to be a very high risk group.

Both a WHZ < −3Z and MUAC < 115 mm must be retained as diagnostic criteria for SAM.

## Additional files

Additional file 1: **Table S1.** Regional grouping of data from countries for meta-analysis. (DOCX 12 kb)

Additional file 2: **Table S2.** Admission diagnostic criteria by country and admission facility. (DOCX 22 kb)

Additional file 3: **Table S3.** Analysis of IPF and OTP patients combined. (DOCX 15 kb)

Additional file 4: **Table S4.** Statistical data on the meta-analysis comparing WHZ-only vs MUAC-only by Region, oedema and treatment facility/program. (DOCX 14 kb)

Additional file 5: **Table S5.** Sensitivity statistics for meta-analysis of WHZ-only vs MUAC-only, by oedema, Region and treatment facility/program. (DOCX 14 kb)

## Abbreviations

CFR: Case fatality rate
IPFs: In-patient treatment facilities
MAM: Moderate acute malnutrition
MUAC: Mid-upper-arm-circumference
NGOs: Non-governmental organisations
OR: Odds ratios
OTPs: Out-patient treatment programs
ROC curve: Receiver operating characteristic curve
RR: Relative risks
SAM: Severe acute malnutrition
SFCs: Supplementary feeding centres
WHZ: Weight-for-height Z-score
