# Supplementary material for: Severely malnourished children with a low weight-for-height have a higher mortality than those with a low mid-upper-arm-circumference: I. Empirical data demonstrates Simpson’s paradox
Source: Nutr J. 2018 Sep 15;17:79. doi: 10.1186/s12937-018-0384-4 (PMC6138885; doi:10.1186/s12937-018-0384-4)
Supplement: Supplementary file 1 — Table S1. Regional grouping of data from countries for meta-analysis. (DOCX 12 kb) [file 12937_2018_384_MOESM1_ESM.docx]

**Additional file 1: Table S1.** Regional grouping of data from countries for meta-analysis

| **Central Africa** | **East Africa** | **Sahel** | **West Africa** |
| --- | --- | --- | --- |
| DRC | Kenya | Chad | Guinee |
| Congo | Tanzania | Mali | Liberia |
| Burundi | Uganda | Somalia | Sierra Leon |
| Rwanda | Ethiopia | South Sudan |  |
| Angola |  | Sudan |  |
|  |  | Niger |  |
