# Supplementary material for: Severely malnourished children with a low weight-for-height have a higher mortality than those with a low mid-upper-arm-circumference: I. Empirical data demonstrates Simpson’s paradox
Source: Nutr J. 2018 Sep 15;17:79. doi: 10.1186/s12937-018-0384-4 (PMC6138885; doi:10.1186/s12937-018-0384-4)
Supplement: Supplementary file 2 — Table S2. Admission diagnostic criteria by country and admission facility. (DOCX 22 kb) [file 12937_2018_384_MOESM2_ESM.docx]

**Additional file 2: Table S2.** Admission diagnostic criteria by country and admission facility

| **Data characteristics** | **M-muac** | | **M-whz** | | **M-both** | | **Kwash** | | **K-muac** | | **K-whz** | | **K-both** | | **Total** |
| --- | --- | --- | --- | --- | --- | --- | --- | --- | --- | --- | --- | --- | --- | --- | --- |
|  | **7 191** | 9.4 | **16 531** | 21.5 | **40 309** | 52.4 | **5 882** | 7.7 | **1 669** | 2.2 | **1 088** | 1.4 | **4 217** | 5.5 | **76 887** |
|  | **n** | % | **n** | % | **n** | % | **n** | % | **n** | % | **n** | % | **n** | % | **n** |
| **IPFs by countries** |  |  |  |  |  |  |  |  |  |  |  |  |  |  |  |
| Angola | 38 | 9.4 | 14 | 3.5 | 205 | 50.7 | 50 | 12.4 | 22 | 5.4 | 6 | 1.5 | 69 | 17.1 | 404 |
| Burundi | 50 | 3.3 | 25 | 1.6 | 300 | 19.6 | 691 | 45.0 | 127 | 8.3 | 96 | 6.3 | 245 | 16.0 | 1 534 |
| Chad | 6 | 1.2 | 32 | 6.6 | 339 | 70.3 | 19 | 3.9 | 10 | 2.1 | 8 | 1.7 | 68 | 14.1 | 482 |
| Congo | 34 | 12.3 | 20 | 7.2 | 171 | 62.0 | 23 | 8.3 | 5 | 1.8 | 5 | 1.8 | 18 | 6.5 | 276 |
| DRC | 189 | 6.7 | 70 | 2.5 | 722 | 25.8 | 1 003 | 35.8 | 358 | 12.8 | 88 | 3.1 | 371 | 13.2 | 2 801 |
| Ethiopia | 16 | 4.1 | 8 | 2.0 | 143 | 36.4 | 94 | 23.9 | 54 | 13.7 | 11 | 2.8 | 67 | 17.0 | 393 |
| Guinea | 27 | 5.5 | 5 | 1.0 | 161 | 33.0 | 146 | 29.9 | 42 | 8.6 | 21 | 4.3 | 86 | 17.6 | 488 |
| Kenya | 9 | 2.0 | 61 | 13.8 | 330 | 74.7 | 8 | 1.8 | 9 | 2.0 | 7 | 1.6 | 18 | 4.1 | 442 |
| Liberia | 33 | 4.9 | 13 | 1.9 | 241 | 35.8 | 175 | 26.0 | 67 | 10.0 | 18 | 2.7 | 126 | 18.7 | 673 |
| Mali | 3 | 1.4 | 19 | 8.6 | 159 | 71.9 | 8 | 3.6 | 7 | 3.2 | 1 | 0.5 | 24 | 10.9 | 221 |
| Rwanda | 203 | 12.4 | 15 | 0.9 | 904 | 55.2 | 163 | 9.9 | 115 | 7.0 | 31 | 1.9 | 208 | 12.7 | 1 639 |
| Sierra Leon | 3 | 2.7 | 3 | 2.7 | 33 | 30.0 | 37 | 33.6 | 11 | 10.0 | 4 | 3.6 | 19 | 17.3 | 110 |
| Somalia | 16 | 5.5 | 4 | 1.4 | 170 | 58.4 | 42 | 14.4 | 9 | 3.1 | 1 | 0.3 | 49 | 16.8 | 291 |
| South Sudan | 0 | 0.0 | 31 | 18.3 | 136 | 80.5 | 0 | 0.0 | 0 | 0.0 | 0 | 0.0 | 2 | 1.2 | 169 |
| Sudan | 39 | 3.2 | 111 | 9.2 | 834 | 69.2 | 63 | 5.2 | 20 | 1.7 | 27 | 2.2 | 112 | 9.3 | 1 206 |
| Tanzania | 13 | 2.3 | 12 | 2.1 | 131 | 23.4 | 270 | 48.3 | 40 | 7.2 | 32 | 5.7 | 61 | 10.9 | 559 |
| Uganda | 80 | 2.5 | 231 | 7.1 | 1 006 | 31.0 | 943 | 29.0 | 242 | 7.5 | 109 | 3.4 | 636 | 19.6 | 3 247 |
| **Total** | **759** | 5.1 | **674** | 4.5 | **5 985** | 40.1 | **3 735** | 25.0 | **1 138** | 7.6 | **465** | 3.1 | **2 179** | 14.6 | **14 935** |
| **OTPs by countries** |  |  |  |  |  |  |  |  |  |  |  |  |  |  |  |
| Chad | 1 | 0.5 | 36 | 17.4 | 158 | 76.3 | 4 | 1.9 | 1 | 0.5 | 0 | 0.0 | 7 | 3.4 | 207 |
| DRC | 112 | 4.0 | 58 | 2.1 | 728 | 26.3 | 1 018 | 36.8 | 386 | 14.0 | 74 | 2.7 | 390 | 14.1 | 2 766 |
| Ethiopia | 46 | 31.1 | 1 | 0.7 | 58 | 39.2 | 31 | 20.9 | 3 | 2.0 | 3 | 2.0 | 6 | 4.1 | 148 |
| Kenya | 1 | 2.1 | 9 | 16.7 | 37 | 77.1 | 0 | 0.0 | 0 | 0.0 | 1 | 2.1 | 1 | 2.1 | 49 |
| Niger | 495 | 1.2 | 9 678 | 24.0 | 27 411 | 68.1 | 526 | 1.3 | 86 | 0.2 | 500 | 1.2 | 1 575 | 3.9 | 40 271 |
| South Sudan | 27 | 3.5 | 278 | 35.9 | 456 | 58.9 | 5 | 0.6 | 4 | 0.5 | 3 | 0.4 | 1 | 0.1 | 774 |
| Uganda | 98 | 8.5 | 66 | 5.7 | 271 | 23.6 | 563 | 49.0 | 51 | 4.4 | 42 | 3.7 | 58 | 5.0 | 1 149 |
| **Total** | **780** | 1.7 | **10 126** | 22.3 | **29 119** | 64.2 | **2 147** | 4.7 | **531** | 1.2 | **623** | 1.4 | **2 038** | 4.5 | **45 364** |
| **SFCs by countries** |  |  |  |  |  |  |  |  |  |  |  |  |  |  |  |
| DRC | 777 | 30.6 | 713 | 28.1 | 1 049 | 41.3 | 0 | 0.0 | 0.0 | 0 | 0 | 0.0 | 0 | 0.0 | 2 539 |
| Kenya | 111 | 9.6 | 941 | 81.6 | 101 | 8.8 | 0 | 0.0 | 0.0 | 0 | 0 | 0.0 | 0 | 0.0 | 1 153 |
| Uganda | 4 764 | 36.9 | 4 077 | 31.6 | 4 055 | 31.4 | 0 | 0.0 | 0.0 | 0 | 0 | 0.0 | 0 | 0.0 | 12 896 |
| **Total** | **5 652** | 34.1 | **5 731** | 34.5 | **5 205** | **31.4** | **0** | **0.0** | 0.0 | **0** | **0** | **0.0** | **0** | **0.0** | **16 588** |

*IPF* In-patient Facility (Hospital. Therapeutic Feeding Center); *OTP* Out-patient treatment program (Home treatment); *SFC* Supplementary Feeding Centre; *M-muac* MUAC <115mm with WHZ ≥-3Z and no oedema (marasmus by MUAC only); *M-whz* WHZ <-3Z with MUAC ≥115mm and no oedema (marasmus by WHZ only); *M-both* MUAC <115mm & WHZ <-3Z and no oedema (marasmus by both diagnostic criteria); *Kwash* nutritional oedema/Kwashiorkor without meeting either MUAC or WHZ criteria; *K-muac* oedematous malnutrition with MUAC < 115mm and WHZ >= -3Z; *K-muac* oedematous malnutrition with MUAC < 115mm and WHZ >= -3Z; *K-whz* oedematous malnutrition with MUAC >= 115mm and WHZ<-3Z; *K-both* oedematous malnutrition with MUAC < 115mm and WHZ <-3Z; *DRC* Democratic Republic of Congo.
