# Supplementary material for: Severely malnourished children with a low weight-for-height have a higher mortality than those with a low mid-upper-arm-circumference: I. Empirical data demonstrates Simpson’s paradox
Source: Nutr J. 2018 Sep 15;17:79. doi: 10.1186/s12937-018-0384-4 (PMC6138885; doi:10.1186/s12937-018-0384-4)
Supplement: Supplementary file 3 — Table S3. Analysis of IPF and OTP patients combined. (DOCX 15 kb) [file 12937_2018_384_MOESM3_ESM.docx]

**Additional file 3:** **Table S3.** Analysis of IPF and OTP patients combined

| **In patients + out patients (IPF + OTP)** | | | | | | |
| --- | --- | --- | --- | --- | --- | --- |
| **Age group** | **Dead** | **Total** | **CFR** | **Relative Risk (95% CI)** | | |
| **6 - 60 m** | # | # | % |  | *lower* | *upper* |
| M-muac | 30 | 1539 | 1.95 | 1.00 |  |  |
| M-whz | 307 | 10799 | 2.84 | 1.46 | *1.01* | *2.11* |
| M-both | 1940 | 35102 | 5.53 | 2.84 | *1.98* | *4.05* |
| Kwash | 337 | 5882 | 5.73 | 2.94 | *2.03* | *4.25* |
| K-muac | 118 | 1669 | 7.07 | 3.63 | *2.44* | *5.38* |
| K-whz | 169 | 1088 | 15.53 | 7.97 | *5.45* | *11.66* |
| K-both | 576 | 4217 | 13.66 | 7.01 | *4.88* | *10.07* |
| **6 - <18 m** | # | # | % |  | *lower* | *upper* |
| M-muac | 13 | 544 | 2.39 | 1.00 |  |  |
| M-whz | 107 | 3 108 | 3.44 | 1.44 | *0.82* | *2.54* |
| M-both | 1 028 | 17 117 | 6.01 | 2.51 | *1.46* | *4.31* |
| Kwash | 33 | 667 | 4.95 | 2.07 | *1.10* | *3.89* |
| K-muac | 20 | 462 | 4.33 | 1.81 | *0.91* | *3.60* |
| K-whz | 26 | 194 | 13.40 | 5.61 | *2.94* | *10.69* |
| K-both | 155 | 1 264 | 12.26 | 5.13 | *2.94* | *8.96* |
| **18 - <36 m** | # | # | % |  | lower | upper |
| M-muac | 10 | 618 | 1.62 | 1.00 |  |  |
| M-whz | 165 | 6 385 | 2.58 | 1.60 | *0.85* | *3.01* |
| M-both | 763 | 15 399 | 4.95 | 3.06 | *1.65* | *5.68* |
| Kwash | 138 | 2 510 | 5.50 | 3.40 | *1.80* | *6.42* |
| K-muac | 43 | 655 | 6.56 | 4.06 | *2.06* | *8.00* |
| K-whz | 83 | 574 | 14.46 | 8.94 | *4.68* | *17.05* |
| K-both | 275 | 2 139 | 12.86 | 7.95 | *4.25* | *14.84* |
| **36 - 60 m** | # | # | % |  | lower | upper |
| M-muac | 7 | 377 | 1.86 | 1.00 |  |  |
| M-whz | 35 | 1 306 | 2.68 | 1.44 | *0.65* | *3.22* |
| M-both | 149 | 2 586 | 5.76 | 3.10 | *1.47* | *6.57* |
| Kwash | 166 | 2705 | 6.14 | 3.31 | *1.56* | *6.99* |
| K-muac | 55 | 552 | 9.96 | 5.37 | *2.47* | *11.65* |
| K-whz | 60 | 320 | 18.75 | 10.10 | *4.68* | *21.78* |
| K-both | 146 | 814 | 17.94 | 9.66 | *4.57* | *20.42* |

Abbreviations are given in table 2.
