# Supplementary material for: Severely malnourished children with a low weight-for-height have a higher mortality than those with a low mid-upper-arm-circumference: I. Empirical data demonstrates Simpson’s paradox
Source: Nutr J. 2018 Sep 15;17:79. doi: 10.1186/s12937-018-0384-4 (PMC6138885; doi:10.1186/s12937-018-0384-4)
Supplement: Supplementary file 4 — Table S4. Statistical data on the meta-analysis comparing WHZ-only vs MUAC-only by Region, oedema and treatment facility/program. (DOCX 14 kb) [file 12937_2018_384_MOESM4_ESM.docx]

**Additional file 4: Table S4.** Statistical data on the meta-analysis comparing WHZ-only vs MUAC-only by Region, oedema and treatment facility/program

| **Group** | **OR** | **LCI 95%** | **HCI 95%** | **Weight (%)** |
| --- | --- | --- | --- | --- |
| **Central Africa** |  |  |  |  |
| Maras IPF DRC | 3.067 | 1.169 | 8.042 | 4.975 |
| Maras SFC DRC | 2.124 | 0.427 | 10.567 | 1.797 |
| Kwash IPF Cent-Africa | 1.277 | 0.668 | 2.442 | 11.015 |
| Kwash IPF DRC | 4.405 | 2.054 | 9.448 | 7.944 |
| *Central Africa subgroup* | 2.298 | 1.504 | 3.511 | 25.730 |
| **East Africa** |  |  |  |  |
| Maras IPF East-Afric | 0.532 | 0.194 | 1.459 | 4.545 |
| Maras OTP East-Afric | 0.773 | 0.159 | 3.763 | 1.846 |
| Maras SFC East-Afric | 0.850 | 0.473 | 1.527 | 13.480 |
| Kwash IPF East-Afric | 1.597 | 0.950 | 2.683 | 17.170 |
| Kwash OTP East-Afric | 3.167 | 0.432 | 23.223 | 1.165 |
| *East Africa subgroup* | 1.106 | 0.781 | 1.566 | 38.208 |
| **Sahel** |  |  |  |  |
| Maras IPF Sahel | 1.529 | 0.233 | 10.045 | 1.305 |
| Maras OTP Sahel | 2.151 | 1.242 | 3.723 | 15.351 |
| Kwash IPF Sahel | 4.654 | 0.893 | 24.241 | 1.698 |
| Kwash OTP Sahel | 2.445 | 1.192 | 5.014 | 8.962 |
| *Sahel subgroup* | 2.315 | 1.534 | 3.494 | 27.316 |
| **West Africa** |  |  |  |  |
| Kwash IPF West-Afric | 1.654 | 0.420 | 6.518 | 2.460 |
| Kwash OTP West-Afric | 17.792 | 7.546 | 41.948 | 6.286 |
| *West Africa subgroup* | 9.122 | 4.409 | 18.875 | 8.746 |
| Pooled | 1.964 | 1.584 | 2.436 | 100 |
| Statistics |  |  |  |  |
| I-squared | 72.156 | 53.230 | 83.424 |  |
| Cochran's Q | 50.281 |  |  |  |
| Chi2. p | 0.000 |  |  |  |

*OR* odds ratio; *Maras* Marasmus; *Kwash* nutritional oedema/Kwashiorkor without meeting either MUAC or WHZ criteria; *IPF* In-patient Facility (Hospital. Therapeutic Feeding Center); *OTP* Out-patient Treatment Program (Home treatment); *SFC* Supplementary Feeding Center; *DRC* Democratic Republic of Congo.
