# Supplementary material for: Severely malnourished children with a low weight-for-height have a higher mortality than those with a low mid-upper-arm-circumference: I. Empirical data demonstrates Simpson’s paradox
Source: Nutr J. 2018 Sep 15;17:79. doi: 10.1186/s12937-018-0384-4 (PMC6138885; doi:10.1186/s12937-018-0384-4)
Supplement: Supplementary file 5 — Table S5. Sensitivity statistics for meta-analysis of WHZ-only vs MUAC-only, by oedema, Region and treatment facility/program. (DOCX 14 kb) [file 12937_2018_384_MOESM5_ESM.docx]

**Additional file 5: Table S5.** Sensitivity statistics for meta-analysis of WHZ-only vs MUAC-only, by oedema, Region and treatment facility/program

| **Excluded group** | **Pooled OR** | **LCI 95%** | **HCI 95%** | **Cochran Q** | **p** | **I 2** | **I 2 LCI 95%** | **I 2 HCI 95%** |
| --- | --- | --- | --- | --- | --- | --- | --- | --- |
| Maras IPF DRC | 1.919 | 1.539 | 2.393 | 49.418 | 0.000 | 73.694 | 55.324 | 84.510 |
| Maras SFC DRC | 1.962 | 1.579 | 2.437 | 50.271 | 0.000 | 74.140 | 56.184 | 84.738 |
| Maras IPF East-Afric | 2.090 | 1.677 | 2.605 | 43.536 | 0.000 | 70.139 | 48.403 | 82.719 |
| Maras OTP East-Afric | 1.999 | 1.609 | 2.484 | 48.922 | 0.000 | 73.427 | 54.808 | 84.375 |
| Maras SFC East-Afric | 2.238 | 1.776 | 2.820 | 41.204 | 0.000 | 68.449 | 45.071 | 81.878 |
| Maras IPF Sahel | 1.971 | 1.587 | 2.447 | 50.212 | 0.000 | 74.110 | 56.125 | 84.722 |
| Maras OTP Sahel | 1.932 | 1.530 | 2.441 | 50.157 | 0.000 | 74.081 | 56.071 | 84.708 |
| Kwash IPF Cent-Afric | 2.072 | 1.650 | 2.602 | 48.375 | 0.000 | 73.127 | 54.228 | 84.222 |
| Kwash IPF DRC | 1.832 | 1.464 | 2.292 | 45.605 | 0.000 | 71.494 | 51.055 | 83.398 |
| Kwash IPF East-Afric | 2.051 | 1.619 | 2.597 | 49.541 | 0.000 | 73.759 | 55.450 | 84.544 |
| Kwash OTP East-Afric | 1.953 | 1.573 | 2.425 | 50.057 | 0.000 | 74.030 | 55.971 | 84.681 |
| Kwash IPF Sahel | 1.935 | 1.558 | 2.404 | 49.213 | 0.000 | 73.584 | 55.113 | 84.455 |
| Kwash OTP Sahel | 1.923 | 1.535 | 2.409 | 49.889 | 0.000 | 73.942 | 55.803 | 84.637 |
| Kwash IPF West-Afric | 1.973 | 1.587 | 2.453 | 50.219 | 0.000 | 74.113 | 56.132 | 84.724 |
| Kwash OTP West-Afric | 1.694 | 1.357 | 2.116 | 23.225 | 0.039 | 44.025 | 0.000 | 70.083 |

*OR* odds ratio; *Maras* Marasmus; *Kwash* nutritional oedema/Kwashiorkor without meeting either MUAC or WHZ criteria; *IPF* In-patient Facility (Hospital. Therapeutic Feeding Center); *OTP* Out-patient Treatment Program (Home treatment); *SFC* Supplementary Feeding Center; *DRC* Democratic Republic of Congo.
